# Defining the Active Fraction of Daptomycin against Methicillin-Resistant *Staphylococcus aureus* (MRSA) Using a Pharmacokinetic and Pharmacodynamic Approach

**DOI:** 10.1371/journal.pone.0156131

**Published:** 2016-06-10

**Authors:** Samira M. Garonzik, Justin R. Lenhard, Alan Forrest, Patricia N. Holden, Jϋrgen B. Bulitta, Brian T. Tsuji

**Affiliations:** 1 Laboratory for Antimicrobial Pharmacodynamics, School of Pharmacy and Pharmaceutical Sciences Buffalo, Buffalo, NY, United States of America; 2 New York State Center of Excellence in Bioinformatics & Life Sciences, University at Buffalo, Buffalo, NY, United States of America; 3 Department of Pharmacotherapy and Experimental Therapeutics, School of Pharmacy, University of North Carolina, Chapel Hill, NC, United States of America; 4 Center for Pharmacometrics and Systems Pharmacology, College of Pharmacy, University of Florida, Orlando, FL, United States of America; University Medical Center Utrecht, NETHERLANDS

## Abstract

Our objective was to study the pharmacodynamics of daptomycin in the presence of varying concentrations of human serum (HS) *in vitro* to quantify the fraction of daptomycin that is ‘active’. Time kill experiments were performed with daptomycin (0 to 256 mg/L) against two MRSA strains at log-phase growth, in the presence of HS (0%, 10%, 30%, 50%, 70%) combined with Mueller-Hinton broth. Daptomycin ≥ 2 mg/L achieved 99.9% kill within 8 h at all HS concentrations; early killing activity was slightly attenuated at higher HS concentrations. After 1 h, bacterial reduction of USA300 upon exposure to daptomycin 4 mg/L ranged from -3.1 to -0.5 log_10_CFU/mL in the presence of 0% to 70% HS, respectively. Bactericidal activity was achieved against both strains at daptomycin ≥ 4 mg/L for all fractions of HS exposure. A mechanism-based mathematical model (MBM) was developed to estimate the active daptomycin fraction at each %HS, comprising 3 bacterial subpopulations differing in daptomycin susceptibility. Time-kill data were fit with this MBM with excellent precision (r^2^ >0.95). The active fraction of daptomycin was estimated to range from 34.6% to 25.2% at HS fractions of 10% to 70%, respectively. Despite the reported low unbound fraction of daptomycin, the impact of protein binding on the activity of daptomycin was modest. The active fraction approach can be utilized to design *in vitro* experiments and to optimize therapeutic regimens of daptomycin in humans.

## Introduction

Significant controversy revolves around the impact of protein binding on antimicrobial activity, particularly for drugs which display a similar affinity to their bacterial target and serum proteins [1, 2]. Since the 1970’s, antibiotic activity has been generally understood to be dependent on free drug concentrations, or inversely related to protein binding [3]. However, there have also been reports of discrepancies between the observed increases in MIC in the presence of proteins (such as albumin), and the difference between total and unbound antibiotic concentrations determined by ultrafiltration [2, 3].

Daptomycin is a cyclic lipopeptide that binds to Gram-positive cell membranes causing rapid depolarization and loss of membrane potential, ultimately leading to rapid bacterial cell death. The novel mechanism of action utilized by daptomycin makes the agent an attractive option for the treatment of infections caused by Gram-positive species such as *Staphylococcus aureus*, particularly strains with decreased susceptibility to vancomycin [4]. Due to daptomycin’s high protein binding of 90–93%, the free fraction of drug found in human plasma is much lower than total plasma concentrations (www.cubicin.com). Several *in vitro* and *in vivo* studies for daptomycin have reported disproportionately higher pharmacodynamic (PD) activity against *S*. *aureus* than is expected based on free-drug concentrations alone [2, 5, 6]. Qualitative *in vitro* reports have also suggested that the presence of protein only impacts the rate of killing, and not the overall activity expressed by daptomycin [2, 6–9].

Based on the discrepancies between daptomycin’s activity and the free fraction of drug, we propose that an ‘active fraction’ of daptomycin may exist that differs from the ‘free-drug fraction’ calculated from reported protein binding values. The active fraction may provide a more appropriate characterization of the extent of protein binding based on bactericidal activity, and guide the translation of *in vitro* activity studies into optimal dosage regimens in humans. Therefore, our objectives were to evaluate the impact of protein binding on the bactericidal activity and time course of killing by daptomycin, and also to develop a mechanism based mathematical model capable of estimating the active fraction of daptomycin in various serum concentrations.

## Materials and Methods

### Ethics Statement

Bacterial isolates used for in vitro investigations were laboratory strains collected from a *S*. *aureus* database. None of the *S*. *aureus* strains utilized in the present study were clinical isolates obtained from patients. Tryptic soy agar with 5% sheep blood (TSA II) was commercially purchased from Fisher Scientific, with specifications available at https://www.fishersci.com/shop/products/bd-bbl-rodac-trypticasesoyagar-5-sheep-blood-tsa-ii-prepared-plated-media/l97759#sthash.Wzi1zwJR.dpuf. Serum was commercially purchased from Sigma Chemical Co. (Lot#098K8712), with specifications available at Sigma-Aldrich.com. Serum was not collected from human patients and no animals were used in the investigation.

### Bacterial Strains

Two bacterial isolates were utilized including i) a vancomycin intermediate *S*. *aureus* strain (VISA), Mu50 (NRS 4, HIP5836, daptomycin MIC = 1.0 mg/L), and ii) an MRSA strain, USA300 (NRS 384, FRP3757, daptomycin MIC = 0.5); both strains were obtained from the Network on Antimicrobial Resistance in *S*. *aureus*.

### Antibiotic, susceptibility testing and medium

Daptomycin analytical grade powder was obtained from Cubist Pharmaceuticals (Lexington, MA). Stock solutions were freshly prepared immediately prior to each experiment. MIC values were determined by broth microdilution in Mueller–Hinton broth (Difco Laboratories, Detroit, Mich.) supplemented with calcium and magnesium (12.5 mg/L; “supplemented Mueller Hinton broth”: SMHB) according to standard methods from the Clinical Laboratory Standards Institute. Human Serum (Lot #098K8712, Sigma Chemical Co., St. Louis, MO) was added to the SMHB to achieve desired concentrations of serum / SMHB. The human serum was heat inactivated at 56°C for 1 hour to inactivate compliment-mediated cell lysis. The final concentrations of human serum / SMHB (v/v) were 0%, 10%, 30%, 50% and 70%. Due to the dependence of daptomycin on calcium for its mechanism of action, the final calcium concentration in each batch of human serum and SMHB was titrated to physiologic conditions (1.1–1.3 mmol/L). Tryptic Soy Agar plates with 5% sheep blood (TSA II) were used to quantify bacterial colony counts (Difco, Detroit, MI).

### Time–kill experiments

Static time kill experiments were performed as previously described [5] in log phase growth against a starting inoculum of 10^6^ CFU/mL. In brief, fresh bacterial colonies were grown overnight then added to SMHB broth to provide a bacterial suspension of approximately 10^8^ CFU/mL; the bacterial suspension was further diluted with SMHB broth to achieve a starting inoculum of approximately 10^6^ CFU/mL. Time kill experiments were conducted for daptomycin against both isolates at concentrations of 0, 0.125 (only for USA300), 0.25, 0.5, 1, 2, 4, 8, 16, 32, 64, and 128 mg/L, in the presence of 0%, 10%, 30%, 50%, and 70% heat inactivated human serum (v/v ratios combined with SHMB) over a period of 24 h. Samples were withdrawn at 0, 1, 2, 4, 8, and 24 hours after dosing, and viable counts were determined by plating 50 μL sample aliquots diluted with saline onto TSA plates with 5% sheep’s blood using an automated spiral dispenser (WASP; Don Whitley Scientific Limited, West Yorkshire, England). Plates were incubated at 35°C for 24 h and viable colonies were quantified using a laser bacteria colony counter (ProtoCOL; Version 2.05.02, Synbiosis, Cambridge, UK). The limit of detection was 10^2^ CFU/mL (equivalent to 5 colonies for an agar plate from an undiluted sample) [10]. Bactericidal activity (99.9% kill) was associated with ≥3.0 log_10_CFU/mL decrease in bacterial density compared to the initial inoculum.

### Pharmacodynamic analyses

To accommodate all available data generated for each concentration tested, and to avoid conclusions based on bacterial counts at a single time point, an integrated pharmacokinetic / pharmacodynamic area measure (log ratio area) was applied to all data as previously described [5]. For each regimen tested, the area under the log_10_CFU/mL versus time curve from 0 to 24 h (AUCFU_0–24_) was calculated via the linear trapezoidal rule for both growth control (AUCFU_growth control_) and drug containing regimens (AUCFU_drug_). The AUCFU_0–24_ was normalized by the AUCFU_0–24_ of the growth control, and the logarithm of this ratio was used to quantify the drug effect (E) as shown in Eq 1A.

$$E={Log}_{10}\left[\frac{{AUCFU}_{drug}}{{AUCFU}_{growth\;control}}\right]$$ (Equation 1a)

Nonlinear fixed effects modeling (version 13; Systat Software Inc., San Jose, CA) was applied to estimate the four parameters of the concentration–effect relationship, which was described according to a Hill type model (Eq 1B).

$$E=E_{0}-\frac{Emax\times {\left[C\right]}^{H}}{{\left[{EC}_{50}\right]}^{H}+{\left[C\right]}^{H}}$$ (Equation 1b)

The dependent variable (E) is the effect described by the log ratio area, E_0_ is the measured effect at zero drug concentration, E_max_ is the maximal drug effect, C is the drug concentration expressed as a multiple of the MIC, EC_50_ is the C:MIC for which there is 50% maximal effect, and H is the Hill or sigmoidicity constant.

### Mechanism Based Mathematical Pharmacodynamic Model

Candidate models were simultaneously fit to all viable count profiles for daptomycin against USA300, which was selected for additional analyses as the most common pulsed-field gel electrophoresis type in the USA. Estimation was performed in NONMEM VI (level 1.2; NONMEM Project Group, Icon Development Solutions, Ellicott City, MD) with the first order conditional estimation method. All residual variability was modeled as described previously [11] using additive and Poisson error models. Model discrimination was based on the curve fits, NONMEM's objective function, and the plausibility of parameter estimates.

### Model for Effect of Human Serum Albumin

The effect of human serum albumin was estimated by calculating the ‘active fraction’ of daptomycin for each level of supplemented human serum. The active fraction is analogous to the free fraction of drug but is a better representation of daptomycin’s activity in the presence of plasma proteins. The f_active_ parameter multiplied by the total concentration of daptomycin yielded the ‘effective’ daptomycin concentration at each concentration of human serum albumin studied (Eq 2). The effect of human serum albumin on bacterial growth rates was also explored.

$$Effective\;Daptomycin\;({DAP}_{EF})=f_{active}(\%\;Human\;Serum)\times Total\;Daptomycin$$ (Equation 2)

### Model for Bacterial Life Cycle

**Fig 1:**
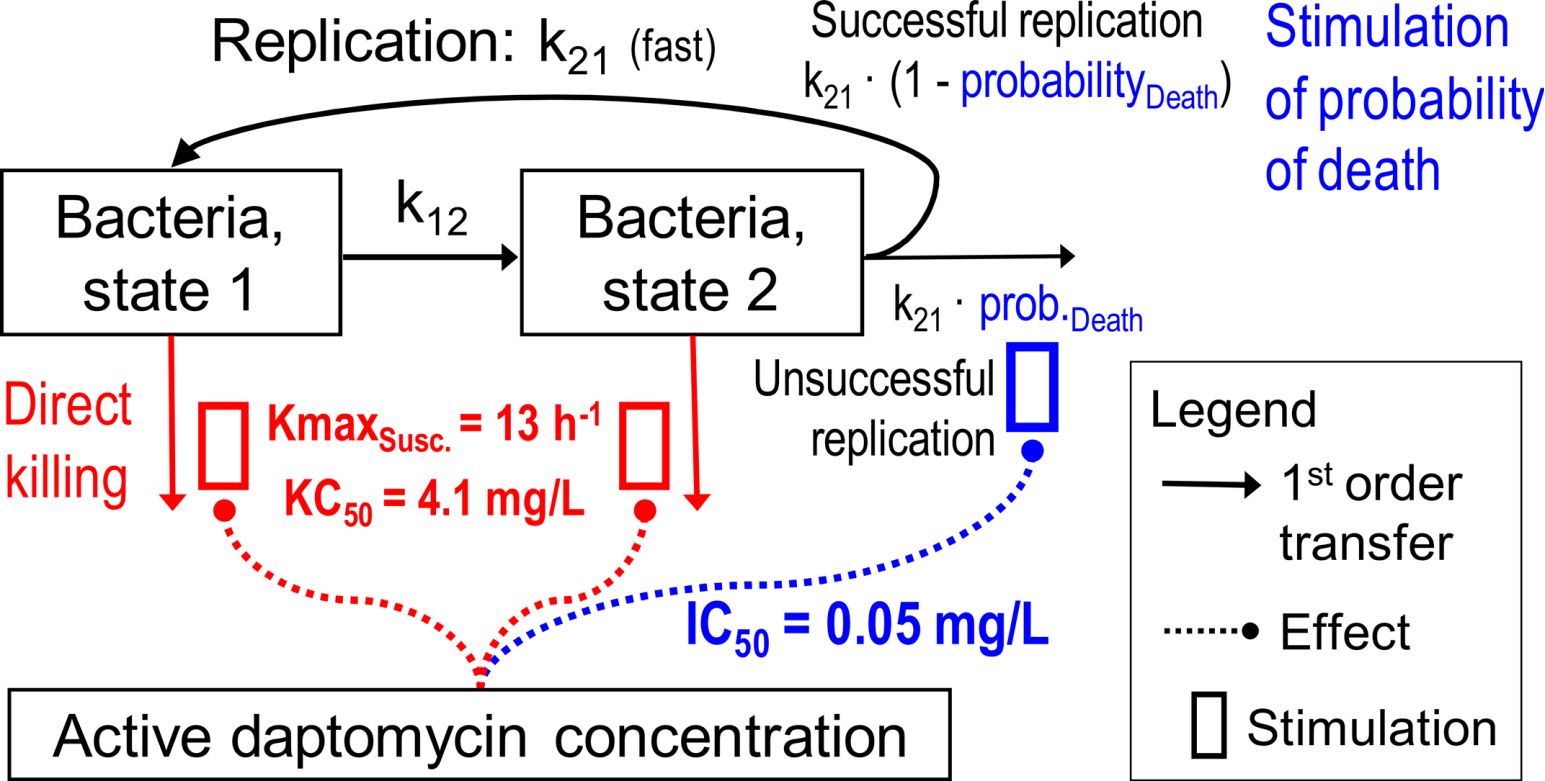
Structural mathematical model for bacterial growth and killing by daptomycin showing both states of the susceptible population (intermediate and ‘resistant’ population not shown).

A simplified life cycle model (Fig 1) was used for bacterial replication in which the bacterial life cycle, for each subpopulation, was described by two states. State1 (S_1_) represents the vegetative state and State2 (S_2_) the replicating state, as described previously [12]. The k_12_ represents the first order transition rate constant from S_1_ to S_2_, while MTT_12_ determines the mean generation time (MTT_12_ = 1/k_12_). Since the doubling was assumed to be very fast, the rate constant for the transition from S_2_ to S_1_ (k_21_) was fixed to 50 h^-1^ [12]. k_21_ is the sum of two processes, namely successful replication and bacterial death. The observed lag time in bacterial growth kinetics was described using Eq 3 [13]. In this equation, k_lag_ represents the first order dissipation of the lag time while β is a sigmoidicity constant.

$$Lag=1-e^{{\left(-k_{lag}∙t\right)}^{\beta }}$$ (Equation 3)

Models with one, two, or three subpopulations of different daptomycin susceptibilities for each strain were considered, as previously described [11, 14–17]. The following equations represent a model with three bacterial subpopulations, susceptible (S_1_ and S_2_), intermediate (I_1_ and I_2_) and resistant (R_1_ and R_2_). Only equations pertaining to one subpopulation are shown. Eq 4 describes the total bacterial inoculum (CFU/ml) at any given time. The plateau reached during growth is modeled using Eq 5, where CFU_m_ represents the maximum achievable CFU/ml count at which the success probability of replication is 50%. Eq 6 was used to model successful replication.

$$CFUtotal=S_{1}+S_{2}+I_{1}+I_{2}+R_{1}+R_{2}$$ (Equation 4)

$$Plateau=(1-\frac{{CFU}_{total}}{{CFU}_{total}+{CFU}_{m}})$$ (Equation 5)

The resistant subpopulation(s) were allowed to have slower growth rates compared to the susceptible subpopulation. Growth rate is decreased at high bacterial burden (Eqs 5 and 6), thus a saturable growth rate function was used to describe the saturation of bacterial growth rate at high bacterial density (Eq 7). Imax_k12_ is the maximal possible inhibition of growth as a function of high bacterial burden and IC50_k12_ is the CFU/mL associated with 50% inhibition of MTTK_12_.

REP=2×Plateau (Equation 6)

$$Growth\;(k_{12es})=Lag\times k_{12}\times (1-\frac{{Imax}_{k12}\times {CFU}_{total}}{{CFU}_{total}+{IC50}_{k12}})$$ (Equation 7)

### Pharmacodynamic Modeling of Daptomycin Activity

Since models with a single killing function were unable to characterize the pharmacodynamic activity of daptomycin, bacterial killing was assumed to be due to stimulation on the probability of death (Eq 8), in addition to direct killing caused by daptomycin (Eq 9). Smax_s_ represents the maximal stimulation of the probability of death for the sensitive subpopulation and SC50 represents the effective daptomycin concentration required to achieve 50% maximal stimulation on the probability of death. Eq 9 determines the fractional inhibition of replication efficiency. Kmax_s_ and KC50_s_ in Eq 10 represent the rate constant for maximal direct killing and the effective daptomycin concentration required to achieve 50% of this effect.

$${STI}_{S}=\frac{{Smax}_{s}\times {DAP}_{EF}}{{DAP}_{EF}+SC50}$$ (Equation 8)

$${IREP}_{S}=1-{STI}_{S}$$ (Equation 9)

$${Kill}_{S}=\frac{{Kmax}_{s}\times {DAP}_{EF}}{{DAP}_{EF}+KC50s}$$ (Equation 10)

Eqs 11 and 12 below represent the differential equations used to model the susceptible subpopulation. In the absence of drug, at low bacterial burden, the replication fraction (REP) approaches 2. At high bacterial burden, REP decreases to 1 which causes a maximum population size. The apparent growth rate constant k_12es_ depends on the bacterial burden. Bacterial killing by daptomycin manifested as stimulation on the probability of death as well as direct killing. The initial conditions (IC) for each equation are provided below:

$$\frac{dS1}{dt}=REP\times k_{21}\times S_{2}\times {IREP}_{s}-k_{12es}\times S_{1}-{Kill}_{s}\times S_{1}\;(IC={CFU}_{0}-I_{1}-R_{1})$$ (Equation 11)

$$\frac{dS1}{dt}=-k_{21}\times S_{2}+k_{12es}\times S_{1}-{Kill}_{s}\times S_{2}\;(IC=0)$$ (Equation 12)

## Results

### Time kill experiments

**Fig 2:**
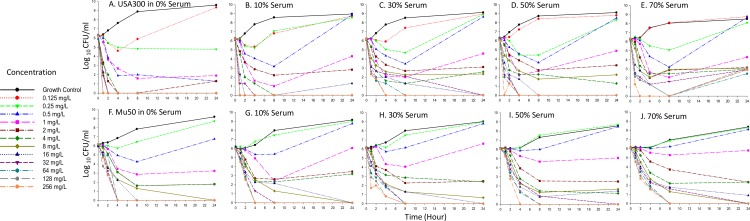
Bacterial killing activity of daptomycin against MRSA USA300 (panels A to E) and VISA Mu50 (panels F to J). Each panel represents increasing v/v ratios of human serum and MHB (0 to 70%).

Fig 2 illustrates the observed bacterial killing curves for both strains at all fractions of heat inactivated human serum studied, highlighting the concentration-dependent killing activity of daptomycin. In general, we observed that as the percent of human serum in broth increased, daptomycin activity was attenuated in a nonlinear fashion. For example, upon exposure to daptomycin 4mg/L, bacterial counts of USA300 after 1 h were -3.1 log_10_CFU/ml (Fig 2A, 0% Human Serum), -1.8 log_10_CFU/ml (Fig 2B, 10% Human Serum), -0.9 log_10_CFU/ml (Fig 2C, 30% Human Serum), -0.8 log_10_CFU/ml (Fig 2D, 50% Human Serum) and -0.5 log_10_CFU/ml (Fig 2E, 70% Human Serum). Similar tendencies were noted for strain Mu50 (Fig 1F–1J). Notably after 24 h, in the absence of serum, bactericidal activity (99.9% kill, -3 log_10_CFU/mL reduction) was achieved against both strains USA300 and Mu50 upon exposure to daptomycin concentrations of >0.25 mg/L and >0.5 mg/L, respectively (Fig 2A and 2F). However, in the presence of all fractions of human serum exposure, higher daptomycin concentrations ≥2mg/L were required to yield bactericidal effects against both strains after 24 h (Fig 2B–2E and 2G–2J). A trend was also noted whereby the time point at which bactericidal activity was reached increased in the presence of higher human serum concentrations for both strains (Fig 2).

**Fig 3:**
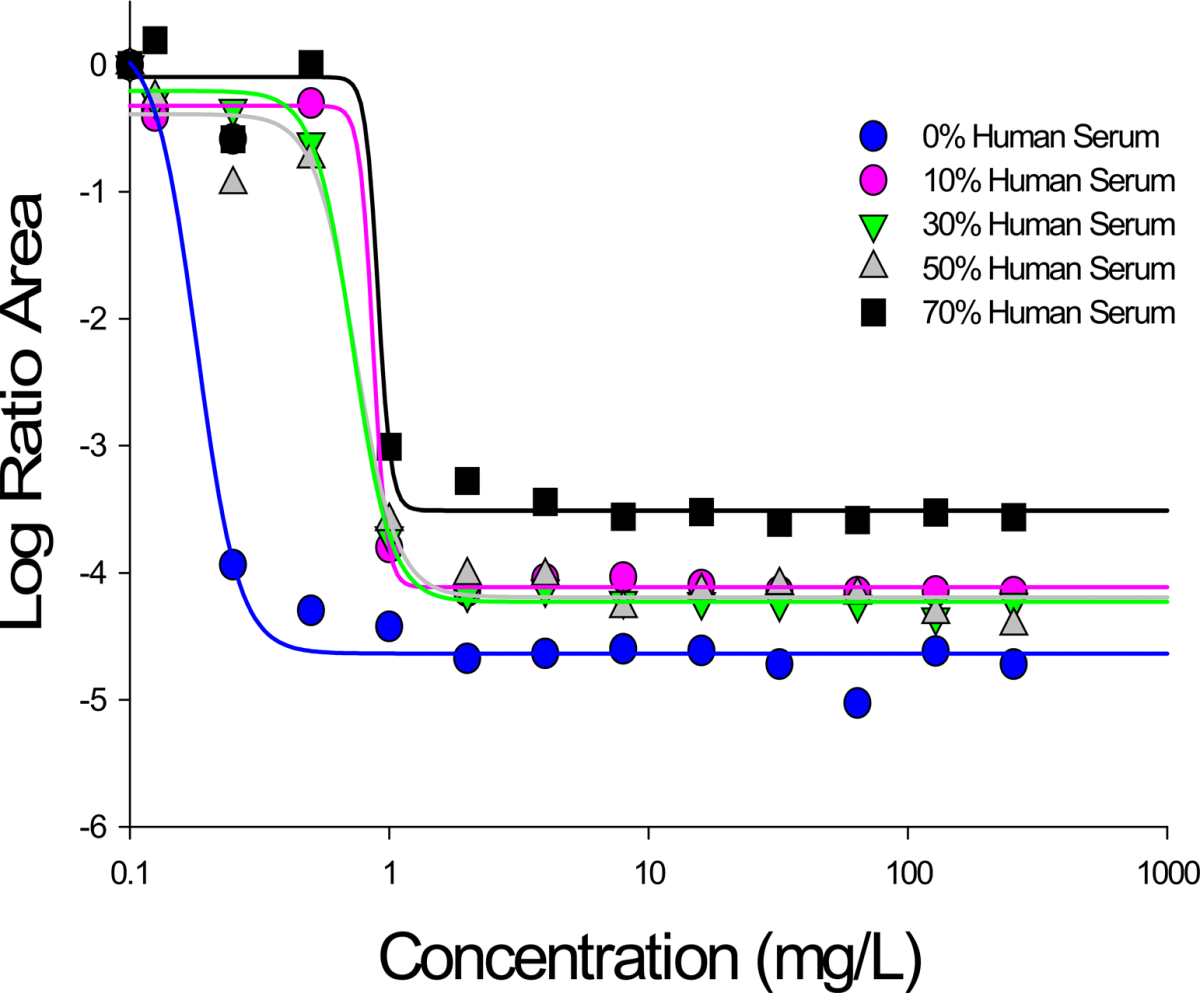
Pharmacodynamic relationship between total daptomycin concentration and the log ratio area at each condition of human serum exposure against USA300. All r^2^ values were >0.97. Similar results were obtained for Mu50.

The antibacterial effect was further quantified as the overall difference in log ratio area for daptomycin *versus* growth control; using this integrated area measure, a gradual reduction in daptomycin activity was quantitatively demonstrated with increasing concentrations of human serum for both strains. For example, when daptomycin 4 mg/L was used against strain USA300, the magnitude of the log ratio area decreased from -4.63 to -3.44 in the presence of 0% to 70% human serum, respectively. In a similar fashion, as the fraction of human serum increased from 0% to 70%, treatment with daptomycin 32 mg/L resulted in an overall area reduction ranging from -4.72 to -3.61, respectively. Identical patterns were noted for strain Mu50. These pharmacodynamic relationships of daptomycin were well fit to a Hill type model (r^2^>0.97) using the log ratio area approach as a measure of effect. Fig 3 visually demonstrates the shift in E_max_ and EC_50_ parameters that occurs with increasing concentrations of human serum, while PD parameters are further presented in Table 1 for both strains.

**Table 1:** Pharmacodynamic Hill-parameters (E_max_, EC_50_, H) for both strains at varying human serum fractions (0% to 70%).

Human Serum	USA300			Mu50		
	E_max_	EC_50_	H	E_max_	EC_50_	H
0%	4.63	0.189	6.19	4.42	0.362	5.82
10%	4.12	0.354	7.17	4.18	0.692	6.34
30%	4.22	0.694	5.29	4.09	0.903	5.98
50%	4.20	0.68	4.07	3.87	1.23	5.63
70%	3.51	0.905	10	3.69	1.66	7.23

### Mechanism based modeling

**Fig 4:**
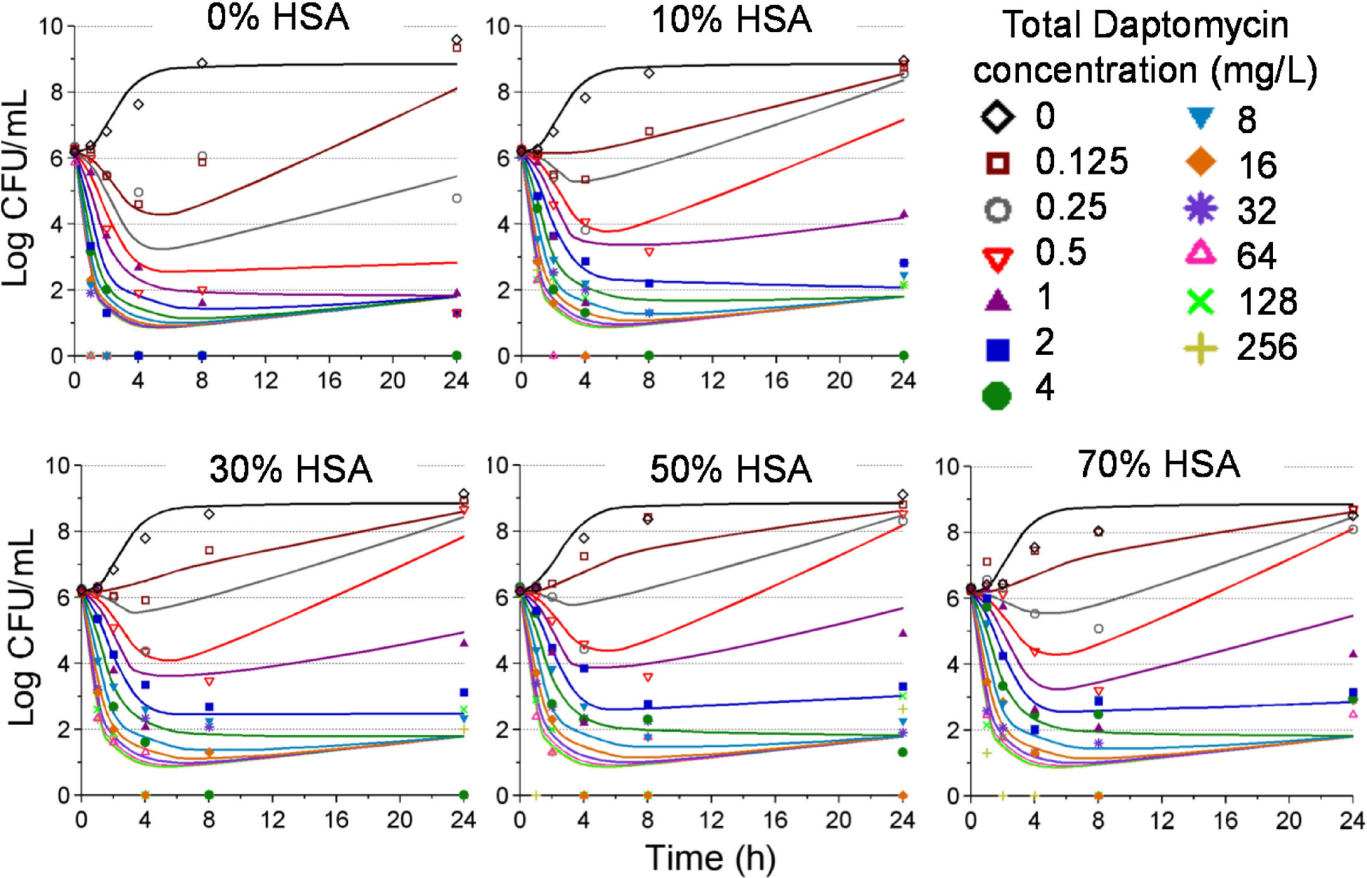
Time kill data (symbols) and model fitted predictions (solid lines) for each condition of human serum exposure for daptomycin against USA300. Each panel represents increasing v/v ratios of human serum and MHB as follows: 0% human serum (panel A), 10% human serum (panel B), 30% human serum (panel C), 50% human serum (panel D) and 70% human serum (panel E).

**Fig 5:**
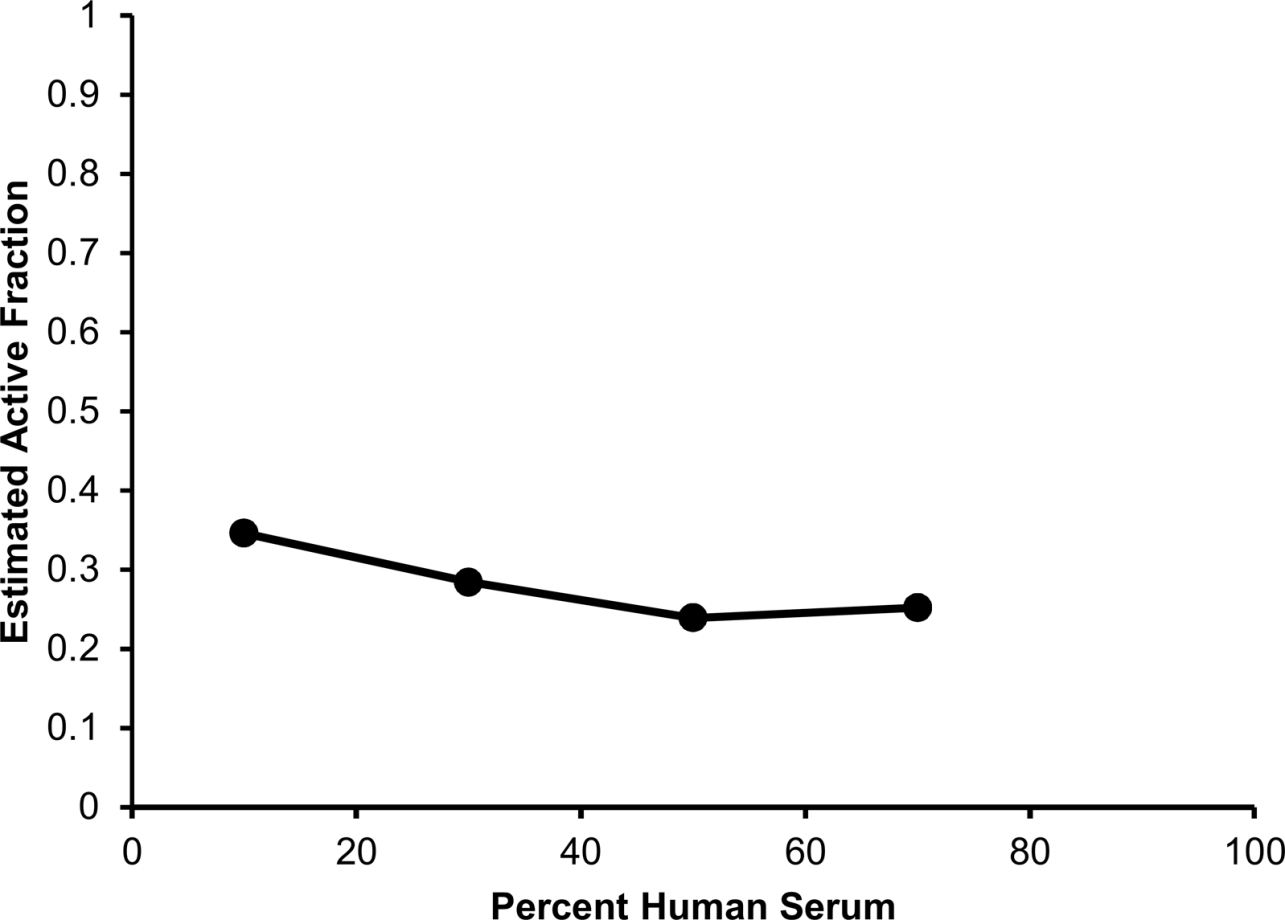
Observed relationship between each concentration of human serum exposure and model estimated active fraction values.

The mechanism based model characterizing the observed pharmacodynamic activity incorporated a mixture model, comprising three bacterial subpopulations differing in daptomycin susceptibility (Fig 1). This model was able to describe all viable count profiles simultaneously with excellent precision, demonstrated by an overall r^2^ value of 0.95 for our population fits. Fig 4 represents the model fitted and observed bacterial counts (log_10_CFU/mL) of USA300 following exposure to each daptomycin concentrations in the presence of increasing human serum fractions. All parameter estimates as well as standard errors are presented in Table 2; relative standard errors were below 41% for all parameters. The mechanism based model estimated a daptomycin active fraction of 35%, 28%, 24% and 25%, in the presence of human serum fractions of 10%, 30%, 50%, and 70%, respectively (Table 1). Fig 5 depicts the relationship of human serum exposure and the model estimated active fraction, demonstrating that at high concentrations of human serum, the active fraction value attains a plateau whereby the pharmacodynamics of daptomycin are not further altered by additional human serum exposure.

**Table 2:** Mechanism based mathematical model parameter descriptions, symbols, units, and estimates (standard error (SE)%), characterizing the pharmacodynamics of daptomycin.

Parameter	Symbol	Units	Estimate (SE%)
Active Fraction at 10% human serum albumin	factive (10%)		0.346 (14%)
Active Fraction at 30% human serum albumin	factive (30%)		0.284 (14%)
Active Fraction at 50% human serum albumin	factive (50%)		0.239 (16%)
Active Fraction at 70% human serum albumin	factive (70%)		0.252 (14%)
Initial inoculum	Log10CFUo		6.22 (0.9%)
Intermediate population as a fraction of initial inoculum	Log10 FR_I_		-3.65 (7.5%)
Resistant population as a fraction of initial inoculum	Log10 FR_r_		-5.67 (3.7%)
MTT for lag time	MTT_lag_	h	75.5 (17%)
Sigmoidicity constant for lag	β		10.0 (0%)
MTT for generation from state 1 to state 2	MTT_K12_	h	20.2 (11%)
Log CFU/mL associated with 50% inhibition of MTTK_12_	Log10IC50_K12_		7.81 (4.4)
Maximum extent of inhibition of K_12_ at high CFU/mL	IMAX_K12_		0.99 (fixed)
Generation from S_2_ to S_1_	K_21_	h^-1^	50.0 (fixed)
CFU/mL count at which success rate of replication is 50%	Log_10_CFU_M_		9.20 (3.7%)
Ratio of K_12i_ / K_12s_	FR_K12i_*		1.00 (fixed)
Ratio of K_12r_ / K_12s_	FR_K12r_		0.0442 (25%)
Maximal stimulation on the probability of death (susceptible population)	Smax_s_^‡^		1 (fixed)
Maximal stimulation on the probability of death(intermediate population)	Smax_i_		0.515 (2.6%)
Maximal stimulation on the probability of death (resistant population)	Smax_r_^*†*^		0 (fixed)
Effective daptomycin concentration required to achieve 50% maximal stimulation on the probability of death	SC50s	mg/L	0.0468 (20%)
Maximal direct killing (susceptible population)	Kmax_s_	h^-1^	14.0 (15%)
Maximal direct killing (intermediate population)	Kmax_i_	h^-1^	1.45 (41%)
Maximal direct killing (resistant population)	Kmax_r_^#^	h^-1^	0 (fixed)
Effective daptomycin concentration required to achieve 50% of maximal direct killing	KC50_s_	mg/L	4.81 (29%)
Additive error for CFU fitted on log scale	*ε*_CFU_		0.558 (11%)
Poisson error	*ε*_Pois_		1.00 (fixed)
Additional Additive error for CFU counts <5	*ε*_Add_		0.250 (fixed)

*FR_K12i_ was estimated close to 1 so this parameter was fixed at 1.

^‡^Smax_s_ was estimated to be very close to 1 so was fixed to 0.99.

^†^Smax_r_ was estimated to be close to zero so was fixed at zero.

^#^Kmax_r_ was estimated close to zero and was thus fixed at zero.

Estimates for SC50_s_ (0.050 mg/L) and KC50_s_ (4.8 mg/L) indicate that low ‘effective’ concentrations of DAP (DAP_EF_) reduced the probability of successful replication, while higher DAP_EF_ concentrations were required to cause direct killing. This observation suggests that the killing function with the greatest pharmacodyamic impact for daptomycin is the inhibition of the probability of successful replication. The key parameter in this function is the SC50_s_.The presence of human serum was not shown to affect the bacterial growth rate (Table 2).

## Discussion

The emergence of MRSA isolates with decreased susceptibility to empiric vancomycin therapy has been increasing steadily [18, 19], particularly in difficult to-treat bloodstream infections. Daptomycin has consequently been highlighted as an alternative treatment option for infections caused by antibiotic resistant Gram positive strains, including MRSA [20]. However, questions are often raised regarding the pharmacodynamics of daptomycin in the presence of protein, owing to the agent’s high protein binding value (90 to 93%, www.cubicin.com). Indeed, the effect of protein binding on antibiotic pharmacodynamics has been a subject of controversy for many years [2, 21, 22]. Although the ultrafiltrate method is traditionally used for protein binding measurements, discrepancies exist between results derived from ultrafiltration *versus* results determined from MIC measurements in the presence and absence of serum proteins [1, 2].

While the effect of protein binding on β-lactams is well documented and predictable, demonstrating a proportionate increase in MIC in the presence of protein relative to the reported protein binding fraction [23, 24], daptomycin does not appear to follow a similar paradigm. Rather, preliminary evidence based on MIC measurements conducted in the presence of physiological concentrations of albumin suggests that extrapolating the free fraction of daptomycin from published protein binding values underestimates the active fraction of drug. [2, 5, 6]. Additionally, *in vitro* time kill experiments and pharmacodynamic models show that although early daptomycin activity was delayed in the presence of albumin, the overall extent of killing was not affected [6, 8, 9]. Similarly, our time kill data for both USA300 and Mu50 strains support the notion of an ‘active fraction’ of drug (Fig 2), where the magnitude of daptomycin’s activity loss was not proportional to the free drug fraction derived from a protein binding level of ~90% (www.cubicin.com). Notably, upon exposure to daptomycin concentrations ≥2 mg/L, bactericidal activity was observed by 24 h against both strains irrespective of the human serum fraction. Delayed killing activity in the presence of higher serum fractions was further highlighted by a reduction in the overall change in log ratio area for daptomycin treated strains *versus* growth control.

Importantly, to the best of our knowledge, a quantitative relationship (or mechanism based model) describing the extent of protein binding and the observed *in vitro* pharmacodynamic activity has yet to be established for daptomycin. Here, we utilized mathematical modeling techniques and pharmacodynamic analyses to quantify the apparent ‘active fraction’ of daptomycin at different concentrations of human serum *in vitro*. Firstly, using a log ratio area approach, daptomycin activity was fit to hill type models for both USA300 and Mu50, from which pharmacodynamic parameters were derived; these parameters demonstrated a clear shift towards lower E_max_ and higher EC_50_ values with increasing human serum fraction, providing an indication of reduced maximal activity and daptomycin potency (Fig 3, Table 1).

Secondly, from our mechanism based pharmacodynamic model, as the human serum fraction increased from 10% to 70%, the estimated active fraction of daptomycin reduced from 34.6% to 25.2%, respectively. These data, suggesting a higher active fraction of daptomycin than that extrapolated using a protein binding value of ~90%, are consistent with other studies which also propose a 28.9 to 51.8% predicted free fraction in varying proportions of human albumin and serum (according to MICs) [8]. This phenomenon may be due to the high relative affinities that daptomycin displays to the site of bactericidal action in *S*. *aureus* compared to plasma protein. For example, daptomycin binds weakly and reversibly to albumin (dissociation constant [K_d_] = 90.3 μmol/L), while irreversible binding is established with the bacterial cell membrane. Jung et. al. recently proposed a two step model, where daptomycin undergoes two conformational changes resulting in rapid bactericidal killing in a calcium dependent manner [25]. During the first step, calcium binds to daptomycin that is weakly bound to the bacterial cytoplasmic membrane, thus promoting lipid interaction. Resultantly, the calcium conjugated daptomycin has an increased amphipathicity and a total decreased charge, which allows for peptide oligomerization leading to the second conformational change; this secondary change enables daptomycin to penetrate deep into the cytoplasmic membrane. Based on the mechanism of irreversible binding, it was postulated that protein bound daptomycin may continue to be available to bind to the cytoplasmic membrane and display antimicrobial activity independent of drug concentration [26]. Taken together with the current findings, these findings suggest that the low reported free fraction of daptomycin alone is not an optimal predictor of pharmacologic effect.

Lastly, the principle of the ‘active fraction’ may not be unique to daptomycin, but may potentially apply to other highly protein bound antimicrobials as well. Similar to the discrepancy between the activity of daptomycin and the free fraction of drug noted in prior investigations, telavancin has displayed in vitro activity that is disproportionately higher than predictions based on unbound drug concentrations [8]. It is therefore likely that in vitro investigations that base telavancin concentrations on the free fraction of drug may be underestimating the activity of telavancin. Further investigations evaluating the performance of highly bound antimicrobials in different concentrations of serum are needed before the model in the present study can be extrapolated to other agents.

An important limitation of the current study is that two daptomycin-susceptible laboratory strains were used in the investigation. While the resistance profiles of the two strains differed for vancomycin, it is unknown whether lipopeptide resistance alters the ‘active fraction’ of daptomycin. Established *S*. *aureus* laboratory strains also possess genotypic differences from contemporary isolates that may result in a discordance in the ‘active fraction’ of daptomycin observed clinically in comparison to the present study. Lastly, USA 300 is a CA-MRSA strain and Mu-50 is a VISA strain originally isolated from a sternal abscess [19]. As neither of the investigated strains were obtained from severe nosocomial infections such endocarditis or bacteremia, caution should be used when extrapolating the results of the current investigation to nosocomial pathogens.

We also acknowledge other potential limitations with this work. Currently, there are no standard methods for incorporating protein into *in vitro* studies to evaluate antimicrobial pharmacodynamics. In the current study, *in vitro* conditions that were established may fail to account for the dynamic physiological interaction between binding of proteins to antimicrobial *in vivo*. Second, daptomycin concentrations were static and the exposure effect relationship, as it relates to AUC/MIC and protein binding, were not explored. Third, although conditions of 100% human serum would best mimic the in vivo situation, this was not studied, since the growth characteristics of MRSA would be hampered at these higher concentrations of serum. Additional studies are warranted in models which account for dynamically changing in vivo conditions with consideration of metabolism, transport processes, and diffusion between compartments in humans [2]. Despite such limitations, we believe that the novel mathematical modeling framework provided here describing the active fraction of daptomycin has potential utility to enhance the design of *in vitro* experiments, and to optimize therapeutic regimens of daptomycin in humans.

## Supporting Information

S1 Data The total bacterial counts (log_10_CFU/mL) for MRSA strain USA300 during exposure to daptomycin in 24 h time-killing experiments performed in the presence of various concentrations of human serum. (PDF)

S2 Data The total bacterial counts (log_10_CFU/mL) for VISA strain Mu50 during exposure to daptomycin in 24 h time-killing experiments performed in the presence of various concentrations of human serum. (PDF)
