# Supplementary material for: Defining the Active Fraction of Daptomycin against Methicillin-Resistant Staphylococcus aureus (MRSA) Using a Pharmacokinetic and Pharmacodynamic Approach
Source: PLoS One. 2016 Jun 10;11(6):e0156131. doi: 10.1371/journal.pone.0156131 (PMC4902307; doi:10.1371/journal.pone.0156131)
Supplement: S1 Data — (PDF) [file pone.0156131.s001.pdf]

		Daptomycin concentration (mg/L)										
	Time (hr)	0	0.5	1	2	4	8	16	32	64	128	256
10% Human Serum	0	6.20	6.22	6.27	6.17	6.26	6.21	6.22	6.04	6.33	6.03	6.24
	1	6.27	6.02	5.87	4.85	4.48	3.56	2.85	2.90	2.30	2.30	2.60
	2	6.81	4.58	3.62	3.64	2.00	2.91	1.60	2.53	0.00	2.38	2.15
	4	7.82	4.06	1.60	2.87	1.30	2.20	0.00	2.00	0.00	1.78	2.00
	8	8.59	3.17	1.00	2.20	0.00	1.30	0.00	1.30	0.00	0.00	0.00
	24	8.97	8.86	4.30	2.82	0.00	2.45	0.00	2.82	0.00	2.15	2.15
30% Human Serum	0	6.24	6.13	6.26	6.16	6.24	6.24	6.24	6.23	6.22	6.12	6.24
	1	6.29	6.14	6.21	5.35	5.35	4.09	3.12	3.20	2.34	2.60	2.30
	2	6.85	5.09	3.79	4.28	2.68	3.30	2.00	2.66	1.60	1.90	1.78
	4	7.80	4.36	2.08	3.36	1.60	2.60	2.00	2.34	1.30	0.00	0.00
	8	8.54	3.47	2.00	2.68	0.00	2.26	1.30	2.08	0.00	0.00	0.00
	24	9.14	8.66	4.60	3.11	0.00	2.34	0.00	0.00	0.00	2.60	2.00
50% Human Serum	0	6.18	6.18	6.29	6.26	6.32	6.16	6.32	6.16	6.29	6.14	6.05
	1	6.29	6.06	6.32	5.60	5.53	4.40	3.58	2.26	2.00	2.90	0.00
	2	7.78	5.30	4.33	4.47	2.78	3.84	2.26	1.78	1.30	2.00	1.30
	4	7.80	4.58	2.20	3.85	2.30	2.70	1.78	0.00	0.00	0.00	0.00
	8	8.36	3.60	2.66	2.76	2.30	1.78	0.00	0.00	0.00	0.00	0.00
	24	9.11	8.52	4.90	3.31	1.30	2.26	0.00	0.00	0.00	0.00	0.00
70% Human Serum	0	6.32	6.26	6.28	6.30	6.23	6.24	6.36	6.28	6.30	6.36	6.32
	1	6.42	6.39	6.26	6.00	5.73	5.25	3.47	2.58	2.45	2.15	1.30
	2	6.44	6.13	5.75	4.26	3.34	2.81	2.85	2.08	1.78	1.78	0.00
	4	7.55	4.38	2.62	2.00	2.45	2.08	1.30	1.30	1.30	1.30	0.00
	8	8.05	3.20	2.08	2.87	2.48	2.95	0.00	1.60	0.00	0.00	0.00
	24	8.52	8.69	4.30	3.15	2.95	3.08	2.90	2.91	2.48	3.08	2.90
