# Supplementary material for: Defining the Active Fraction of Daptomycin against Methicillin-Resistant Staphylococcus aureus (MRSA) Using a Pharmacokinetic and Pharmacodynamic Approach
Source: PLoS One. 2016 Jun 10;11(6):e0156131. doi: 10.1371/journal.pone.0156131 (PMC4902307; doi:10.1371/journal.pone.0156131)
Supplement: S2 Data — (PDF) [file pone.0156131.s002.pdf]

		Daptomycin Concentration (mg/L)										
	Time (hr)	0	0.5	1	2	4	8	16	32	64	128	256
10% Human Serum	0	5.93	5.95	6.00	5.98	5.93	5.93	5.94	6.00	5.96	5.89	5.93
	1	6.15	5.97	6.00	5.91	5.69	5.47	5.28	4.90	4.78	4.57	4.46
	2	6.20	5.91	5.81	5.12	4.58	4.01	3.54	3.31	2.90	2.75	2.53
	4	6.88	5.34	4.90	3.11	3.02	2.56	2.20	1.30	0.00	0.00	1.60
	8	7.98	5.37	2.41	1.60	1.30	1.30	0.00	0.00	0.00	0.00	0.00
	24	9.09	8.75	6.06	1.78	0.00	0.00	0.00	0.00	0.00	0.00	0.00
30% Human Serum	0	6.08	5.99	6.09	6.11	6.01	6.06	6.13	5.96	6.09	6.10	6.06
	1	6.22	5.99	6.03	5.98	5.79	5.53	3.49	4.70	2.62	2.15	0.00
	2	6.34	5.98	5.64	3.53	2.96	2.38	3.58	3.06	2.76	2.30	1.90
	4	7.03	5.86	4.41	3.79	-	1.30	1.30	1.60	1.60	0.00	0.00
	8	8.32	6.86	4.47	1.78	3.51	0.00	0.00	0.00	0.00	0.00	1.30
	24	9.15	8.68	7.56	2.87	3.53	1.30	0.00	0.00	0.00	0.00	0.00
50% Human Serum	0	6.22	6.18	6.15	6.09	6.08	6.09	6.06	6.12	6.17	6.11	6.06
	1	6.24	6.07	6.22	6.15	6.05	6.08	5.69	5.53	5.48	5.40	5.15
	2	6.35	6.06	5.87	5.75	5.23	4.69	4.18	3.68	3.34	2.82	2.45
	4	6.81	5.85	5.15	3.91	2.87	2.34	1.90	2.85	1.30	1.30	0.00
	8	7.74	5.84	4.64	2.38	1.30	0.00	1.60	0.00	0.00	0.00	0.00
	24	9.14	8.33	4.81	2.41	2.38	1.30	1.60	0.00	0.00	0.00	0.00
70% Human Serum	0	6.27	6.05	6.18	6.09	6.19	6.18	6.11	6.06	6.11	6.10	6.10
	1	6.33	6.11	6.13	6.04	6.09	5.82	5.58	5.14	4.86	4.34	4.26
	2	6.57	6.15	5.97	5.85	5.20	4.41	3.81	3.43	3.19	2.76	2.48
	4	7.21	6.11	5.67	4.68	3.13	2.15	1.78	1.90	1.30	1.78	1.30
	8	7.93	6.46	5.49	3.88	2.20	1.60	1.78	1.60	0.00	0.00	0.00
	24	9.21	8.14	6.61	2.41	2.34	0.00	1.78	0.00	0.00	0.00	0.00
